# Functional Outcome of Distal Humerus Fractures Treated With Open Reduction and Internal Fixation With Bicolumnar Plating in a Tertiary Care Setting

**DOI:** 10.7759/cureus.33540

**Published:** 2023-01-09

**Authors:** U Jagadish, Vinod Kumar K, Arun H Shanthappa

**Affiliations:** 1 Orthopaedics, Sri Devaraj Urs Medical College, Kolar, IND; 2 Orthopaedics and Trauma, Sri Devaraj Urs Medical College, Kolar, IND

**Keywords:** orthogonal plate fixation, bicolumnar plating, mayo elbow performance scoring index, elbow range of motion, distal humerus fractures

## Abstract

Introduction

Fractures of the distal humerus in the adult comprise approximately one-third of all humeral fractures. Over the past 20 years, nonoperative treatment for these fractures has been substituted by anatomic reduction and internal fixation based on the Association for Osteosynthesis (AO)/Association for the Study of Internal Fixation (ASIF) philosophy of plate fixation which resulted in early mobilization and superior performance. Pre-contoured, anatomically designed locking plates are anticipated to offer sufficient stability, permit early elbow range of motion, and safeguard the soft tissue. In comparison to any other joint, the elbow's good anatomical alignment, perfect stability, and early mobilization principles are of utmost significance.

Methodology

A hospital-based consecutive case series of distal humerus fracture patients managed surgically with bicolumnar plating at R.L. Jalappa Hospital Centre, from June 2021 to June 2022 was chosen. Patients were clinically assessed by measuring the range of motion of the elbow with a goniometer. A six-week post-operative review was the first one. Routine checkups were scheduled every four weeks up until there was evidence of fracture consolidation radiologically. Clinically Mayo Elbow Performance score (MEPS) was analyzed at the end of six-month follow-up and tabulated. Institutional Ethical committee permission was taken prior to the study.

Results

In the study, 47% of cases had an Excellent MEPS followed by 33% of patients having a good MEPS and 13% having a Fair MEPS rating. Only 7% of patients had poor MEPS. Among the patients, 33.3% had 90 MEPS followed by 16.6% cases had 85 MEPS. Only 2 patients had 55 MEPS in the study. The fracture pattern configuration based on AO classification in our study was C2>C1>C3>A2.3=A3.3.

Conclusion

Due to an increase in road traffic accidents, complicated distal humeral fractures are becoming more common among younger people. In terms of stability and arc of motion, excellent to good functional outcomes were attained in around 80% of the study group. Because of the extremely stable build system produced by parallel plating, there have been no reported instances of implant failure or non-union.

## Introduction

In adults, distal humerus fractures account for about 30% of all humeral fractures and 2% of all fractures [1]. Managing such fractures is quite challenging due to the intricate anatomy of the elbow joint, the neurovascular system in the area, and the lack of soft tissue [2]. The major goal of treating distal humerus fractures is to keep the elbow stable while still allowing for an acceptable functional range of motion. So, it is critical to determine whether fracture repair produces a stable mobile joint.

Over the past 20 years, nonoperative treatment for these fractures has been replaced by anatomic reduction and internal fixation based on the Association for Osteosynthesis (AO)/Association for the Study of Internal Fixation (ASIF) principle of parallel plate fixation which resulted in the mobilization and better performance. The risks of functional impairment and deformity after conservative treatment for these distal intra-articular humeral fractures are relatively high. The prolonged time duration for internal healing may be challenging to achieve due to the severity of these intra-articular fractures and osteoporosis in the case of elderly patients [3,4].

In distal humerus fractures, several different forms of plating are utilized. When it comes to difficult fractures, the typical compression plate/locking compression plate does not offer enough stability [5,6]. The standard procedure for reliable fixing of small-sized distal pieces is double plating in parallel or orthogonally. However, its benefit is constrained by the potential for significant soft tissue amputation, longer recovery times, and increased infection and nonunion risks [7,8]. Pre-contoured, anatomically designed locking plates are anticipated to offer sufficient stability, permit early elbow range of motion, and safeguard the soft tissue [9].

In comparison to any other joint, the elbow's good anatomical alignment, perfect stability, and early mobilization principles are of utmost significance. Most current recommendations for treating distal humeral fractures involve using plates and screws in an open reduction and internal fixation (ORIF) procedure. An ORIF of the fracture enables the surgeon to realign the fracture fragments anatomically and to do early range-of-motion (ROM) exercises that could help the elbow regain functional range of motion after surgery [10,11].

Internal fixation techniques have changed throughout time in an effort to best restore the distal humerus's anatomical alignment. The literature has lately discussed where anatomically to position the plates on the distal humerus, with the majority of writers now advising at least two plates be used to offer appropriate stability and allow for proper restoration of anatomy. The recommendations made by the AO/ASIF group, which call for the placement of two plates at a 90° angle to one another (orthogonal/perpendicular/90°/90° plating), have been the gold standard for fixing distal humeral fractures up to this point. As a result of implant failure happening if deployed too soon, many researchers have reported poor outcomes in 20% to 25% of patients using these fixation approaches [12,13].

There was a paucity of documented literature determining the functional outcome of distal humeral fractures treated with ORIF with bicolumnar plating. So, the present study was conducted.

## Materials and methods

**Figure 1:**
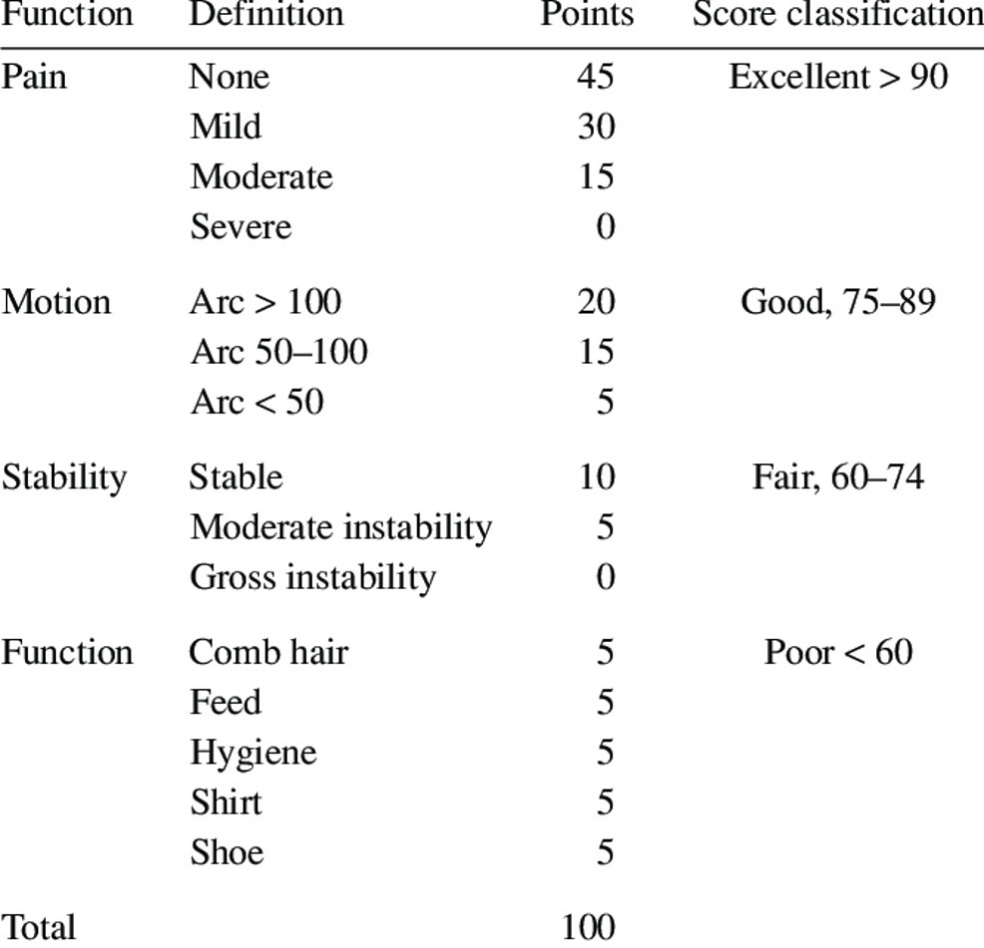
Description of MEPS and its points MEPS - Mayo Elbow Performance Score

A hospital-based consecutive case series of distal humerus fracture patients managed surgically with bicolumnar plating at R.L. Jalappa Hospital Centre, from June 2021 to June 2022 after obtaining permission from the Institutional Ethics Committee of Sri Devaraj Urs Medical College (No. DMC/KLR/IEC/181/2022-23). All patients who had distal humerus fractures treated with bicolumnar plating were included in the study. Patients who had sustained additional fractures over the ipsilateral limb or Gustilo-Anderson type II/III open fractures, any pathological fractures, or pre-existing deformity and Pre-existing infection were excluded. Patients were clinically assessed by measuring the range of motion of the elbow with a goniometer. The first post-operative review was at six weeks. Subsequent appointments were at every 4-6 weeks until fracture consolidation was observed. Clinically Mayo Elbow Performance score (Figure 1) was analyzed at the end of six months follow-up and tabulated.

A sample size of 30 patients was calculated based on previous study conducted by Mahajan et al. considering MEPS points and using the callus formation radiologically.

Data entry and analysis

Data were entered into an MS Excel sheet and analyzed by using IBM SPSS version 21.0 software (IBM Corp., Armonk, NY, USA). Qualitative data are described as frequency and percentages.

## Results

The majority (36.6%) of participants were aged more than 50 years. A total of eight (26.6%) patients belonged to the 41-50 years of age group and six (20%) belonged to the 21-30 years of age group. Five (16.8%) of the participants belonged to the 31-40 years of age group as mentioned in Table 1.

**Table 1:** Age-wise distribution of study participants (n=30)

Age group	Frequency (%)
21-30 years	6(20)
31-40 years	5(16.8)
41-50 years	8(26.6)
> 50 years	11(36.6)

**Figure 2:**
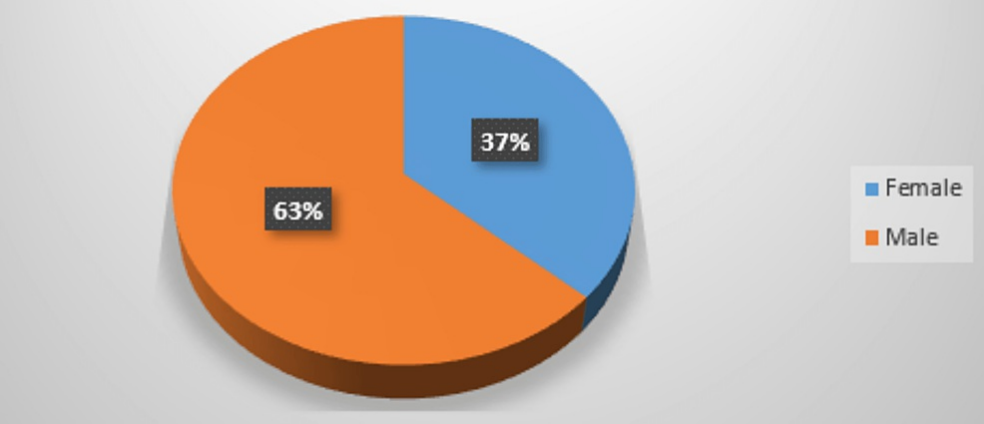
Gender wise distribution among study participants

In the study, 63% were males and 37% were females as mentioned in Figure 2.

**Figure 3:**
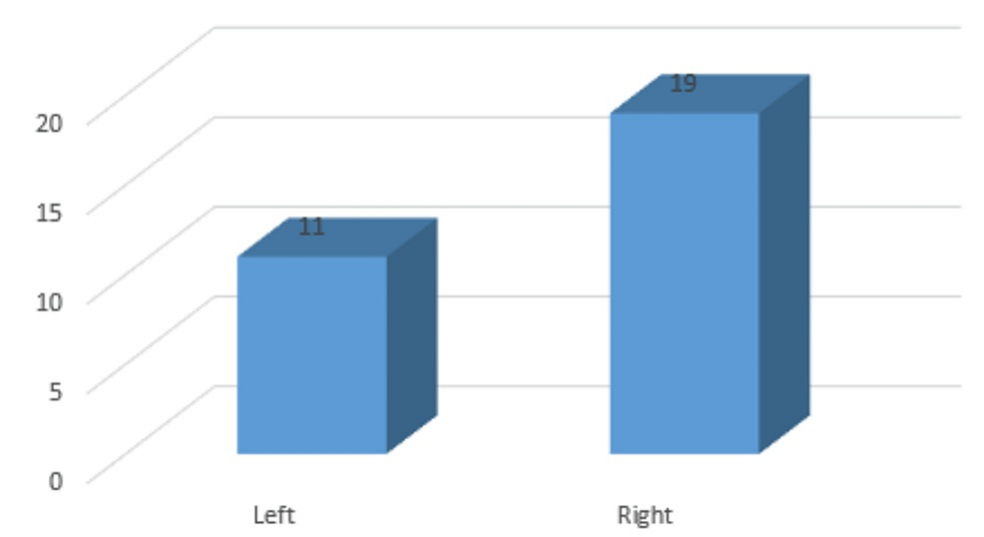
Affected side of limb among study participants

Among the study participants, 11 were affected on the left side while the remaining 19 were affected on the right side as mentioned in Figure 3.

Most of the patients 16 (53.4%) were injured due to motor vehicle accidents. A total of 12 (40%) patients were injured by falls from height and the remaining two (6.6%) cases were injured due to assault as mentioned in Table 2.

**Table 2:** Distribution of mode of injury seen in study participants (n=30)

Mode of Injury	Frequency (%)
Assault	2(6.6)
Fall	12(40)
Motor vehicle accident	16(53.4)

Out of the total, 13 (43.3%) patients had a C2 type AO fracture pattern followed by 11 (36.7%) cases that had C1 type AO fractures. A total of four (13.4%) had C3 type AO fracture patterns as mentioned in Table 3.

**Table 3:** Distribution of AO type among study participants (n=30)

AO type	Frequency (%)
A2.3	1(3.3)
A3.3	1(3.3)
C1	11(36.7)
C2	13(43.3)
C3	4(13.4)

**Figure 4:**
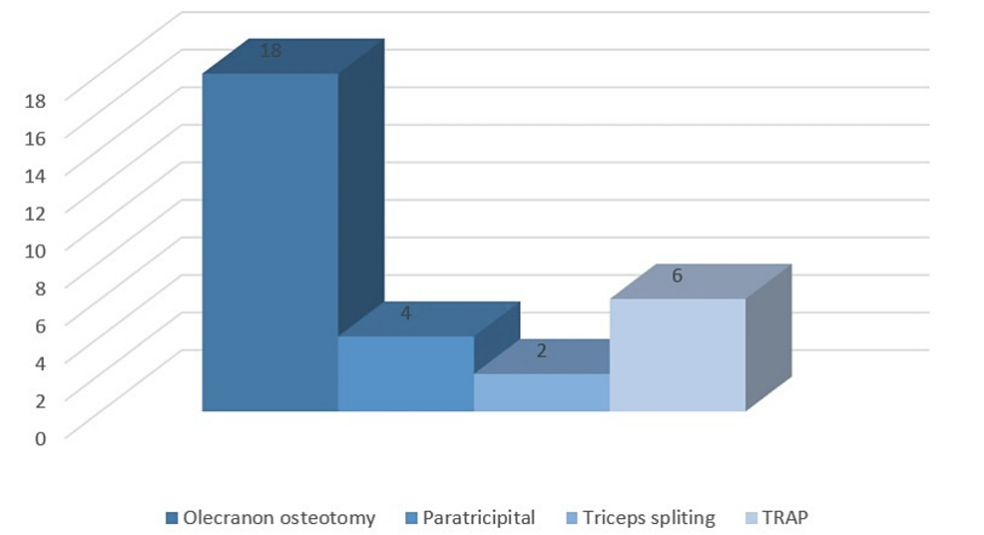
Distribution of approach taken in study participants

Most of the patients had been approached by the Olecranon osteotomy procedure followed by Triceps-reflecting anconeus pedicle (TRAP) approach and Paratricipital. While remaining two patients were approached by triceps splitting as mentioned in Figure 4.

Among the patients, 30.7% patients had associated injury of right superior pubic rami fracture, and another 30.7% patients had associated injuries as mentioned in Table 4.

**Table 4:** Distribution of associated injury among study participants (n=13)

Associated Injury	Frequency (%)
Contralateral shaft of Humerus fracture	1(7.6)
Contralateral Distal Radius fracture	4(13.3)
Head injury	1(7.6)
Contralateral Median Nerve palsy	1(7.6)
Contralateral Metacarpal fracture	1(7.6)
Right superior pubic rami fracture	4(30.7)
Right side fracture of femur shaft	1(7.6)

**Figure 5:**
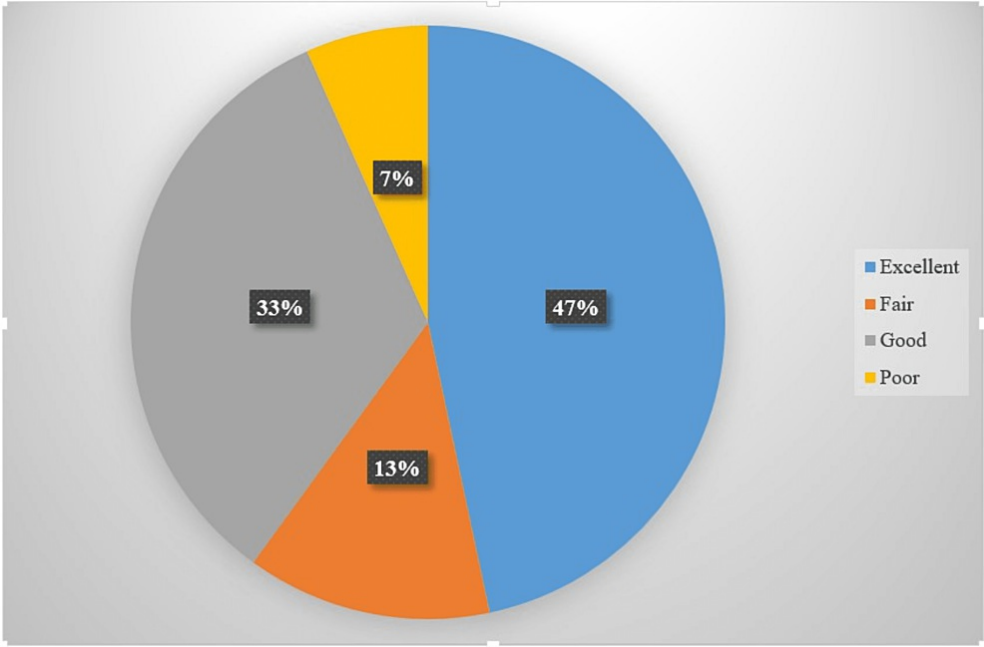
Distribution of MEPS among study participants MEPS- Mayo Elbow Performance Score

In the study, 47% of cases had an Excellent MEPS followed by 33% of patients who had a good MEPS and 13% had fair MEPS. Only 7% of patients had poor MEPS as mentioned in Figure 5.

Among the patients, 10 (33.3%) had 90 MEPS points followed by five (16.6%) cases had 85 MEPS points. Only two (6.6%) patients had 55 MEPS points in the study as mentioned in Table 5.

**Table 5:** Distribution of MEPS points among study participants (n=30) MEPS - Mayo Elbow Performance Score

MEPS	Frequency (%)
55	2(6.6)
70	4(13.3)
80	5(16.6)
85	5(16.6)
90	10(33.3)
95	4(13.3)

In the study, four patients had paraesthesia in the ulnar nerve sensory area. While two patients had developed complications due to hardware prominence and two anther patients had developed superficial infections. Only in two patients, there was a decrease in ROM due to heterotopic ossification as mentioned in Table 6.

**Table 6:** Distribution of Complications among study participants (n=10)

Complications	Frequency (%)
Paraesthesia in ulnar nerve sensory area	4(34)
Hardware Prominence	2(22)
Decreased ROM due to heterotrophic Ossification	2(22)
Superficial Infections	2(22)

## Discussion

In our study, the largest subgroup (36.6%) of participants were aged more than 50 years. A total of eight (26.6%) patients belonged to the 41-50 years of age group and six (20%) belonged to the 21-30 years of age group. In the study, 63% were males and 37% were females.

While in the study of Mahajan et al. [13], 20 of the 35 cases were female, and 15 were male. A history of falls and road traffic accidents (RTA) was present in 60% and 40% of cases, respectively. The age range with the highest prevalence was >60 years (37.14%), followed by 31-45 years (34.29%) as mentioned in Table 7.

**Table 7:** Comparison of age group of study participants with other study

Age group	In present study	In study of Mahajan et al. [13]
21-30 years	20%	21.5%
31-40 years	16.8%	34.29%
41-50 years	26.6%	7.07%
> 50 years	36.6%	37.14%

Most of the patients had approached the olecranon Osteotomy procedure followed by Trap and Paratricipital. While remaining two patients were approached by triceps splitting. Among the patients, 30.7% patients had associated injury of right superior pubic rami fracture, and another 30.7% patients had associated injury of distal radius fracture.

In the study, 47% of cases had an Excellent MEPS followed by 33% of patients having a good MEPS and 13% having a Fair MEPS. Only 7% of patients had poor MEPS. Among the patients, 33.3% had 90 MEPS points followed by 16.6% cases had 85 MEPS points. Only two patients had 55 MEPS points in the study. In the study, four patients had paraesthesia in the ulnar nerve sensory area. While two patients had developed complications due to hardware prominence and two another patients had developed superficial infections. Only in two patients, there was a decrease in ROM due to heterotopic ossification.

While in the study of Virani et al. [14], there were a total of 11 instances of type 13-C1, 23 cases of type 13-C2, and seven cases of type 13-C3. Of the patients involved, 14 were female and 27 were male. The follow-up period ranged from 26 to 82 months, with a mean of 38 months. Implant removal and implant prominence had been performed on two patients. Radiological evidence of elbow arthritis was present in three cases. The olecranon osteotomies were without problems, and two patients required secondary surgery to remove a mass of heterotopic ossification. In the present study, 36.7% of patients had C1 fractures, 43.3% had C2 fractures, 13.4% had C3 fractures as mentioned in Tables 8, 9.

**Table 8:** Comparison of AO classification of distal humeral fractures among various study

	C1	C2	C3
In present study	36.7%	43.3%	13.4%
Virani et al. [14]	26.8%	56.09%	17.07%

**Table 9:** Comparison of gender among various studies

	Male	Female
In present study	63%	37%
Virani et al. [14]	65.8%	34.2%
Ditsios et al. [15]	60.7%	29.3%

In the present study, a total of 11 cases were affected on the left side while the remaining 19 were affected on the right side. Most of the patients (53.4%) were injured due to motor vehicle accidents. A total of 12 patients were injured by a fall down and the remaining 6.6% of cases have injured the cause of the assault. 

In the study [15], AO type C distal humeral fractures were treated in 26 patients in a row using either parallel (Group B: 11 patients; mean age 56.5 years, range 17-86) or orthogonal (Group A: 15 patients; mean age 53.5 years, range 21-96) plate fixation. The patients were evaluated radiographically, clinically, and using the Mayo Elbow Performance Index. There were 24 patients (10 from group B and 14 from group A) who could be followed up. In comparison to group B, group A had a mean follow-up of 33 months versus 48.8 months. In group A, seven elbows had grades of excellent, five of good, one of fair, and one of poor according to MEPS, whereas in group B, six elbows received grades of excellent and four of good.

Limitations

A multi-centric study with a larger sample size can produce better results, even though the current study met the minimum sample size requirement. Our study's follow-up period was incredibly brief (six months duration from the time of surgery). Further research is required to understand the functional outcome of the operated patient over a long period of time.

## Conclusions

Due to an increase in road traffic accidents, complicated distal humeral fractures are becoming more common among younger people. A superior functional outcome is made possible by the system's complete stability, which enables early post-operative rehabilitation. In terms of stability and arc of motion, excellent to good functional outcomes were attained in around 80% of the study group. Because of the extremely stable build system produced by parallel plating, there have been no reported instances of implant failure or non-union. Though it may look like a variation on the usual plate placement technique, it is actually a completely unique idea that offers more stability in comminuted intraarticular fractures. When its principles are properly followed, parallel plating can be an effective approach for the internal treatment of these difficult fractures.
